# Supplementary material for: Comparative multiomics analysis of cell physiological state after culture in a basket bioreactor
Source: Sci Rep. 2022 Nov 23;12:20161. doi: 10.1038/s41598-022-24687-4 (PMC9686226; doi:10.1038/s41598-022-24687-4)
Supplement: Supplementary file 1 — Supplementary Information 1. [file 41598_2022_24687_MOESM1_ESM.zip › raw data/Transcriptomic raw data/5.Differential/1.deglist/TE1vsTC1/TE1vsTC1_volcano.pdf]

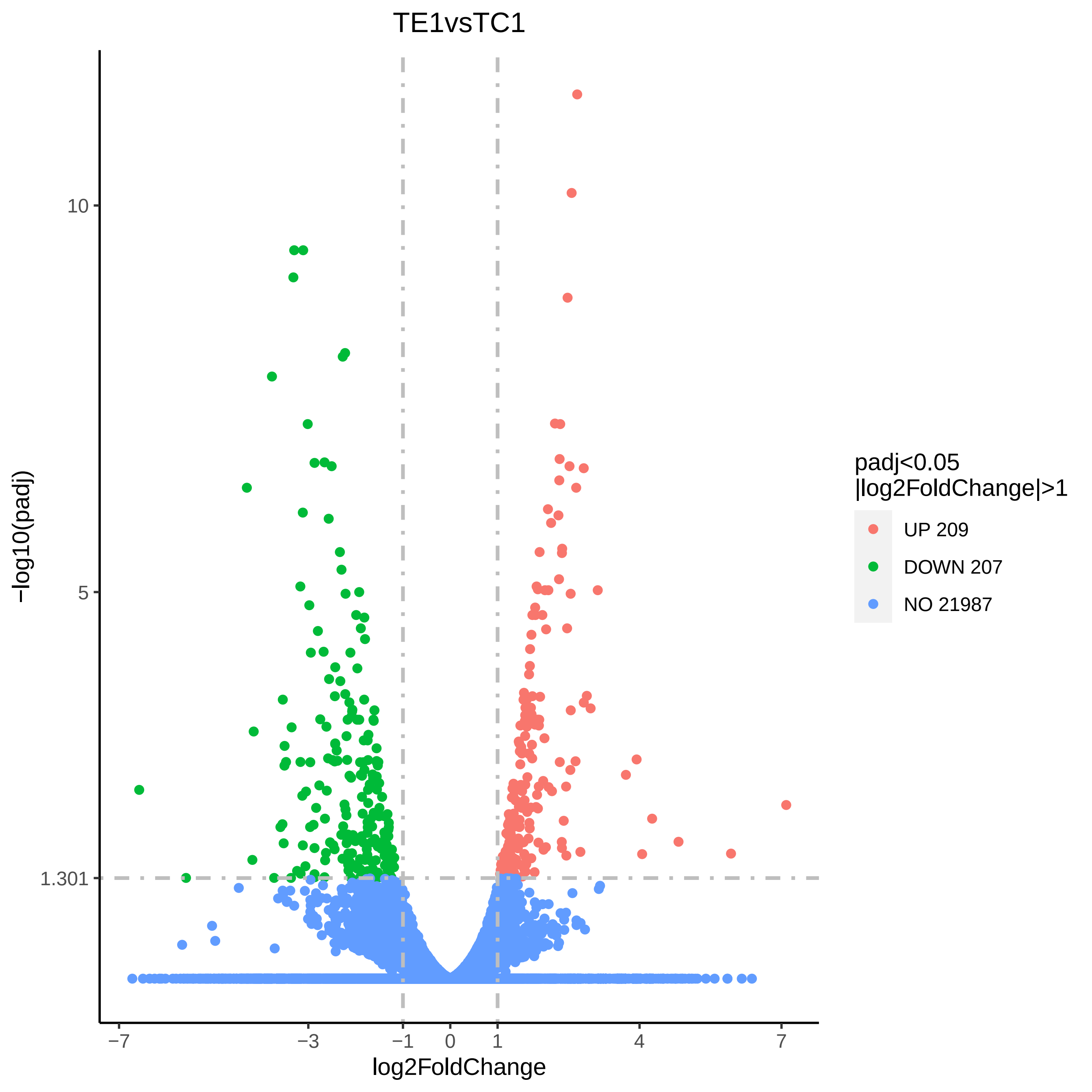

TE1vsTC1
−log10(padj)
10
5
1.301
−7
−3
−1
0
1
4
7
log2FoldChange
padj<0.05
|log2FoldChange|>1
UP 209
DOWN 207
NO 21987
